# Reduced long-term periprosthetic fracture rates with composite beam versus polished tapered stems in cemented hip arthroplasty: a ten-year observational study

**DOI:** 10.1302/2633-1462.73.BJO-2025-0350

**Published:** 2026-03-06

**Authors:** Viktor Mili-Schmidt, Martin Magnéli, Kartik Logishetty, Olof Sköldenberg, Michael Axenhus

**Affiliations:** 1 Department of Clinical Sciences at Danderyd Hospital, Division of Orthopedics, Karolinska Institutet, Stockholm, Sweden; 2 Imperial College Healthcare NHS Trust, London, UK

**Keywords:** Adverse events, Cemented hip arthroplasty, Periprosthetic femoral fractures, Prosthetic joint infections, Stem design, cemented hip arthroplasties, periprosthetic fractures, Composite beam, Femoral components, revision surgeries, periprosthetic joint infection, Anesthesiologists, cognitive dysfunction, hip arthroplasties, hemiarthroplasties

## Abstract

**Aims:**

This study evaluates the effect of an orthopaedic department’s full transition from the use of a cemented polished tapered stem (PTS) to a cemented composite beam femoral component (CB) on periprosthetic fracture rates up to ten years after primary surgery.

**Methods:**

A ten-year, prospective observational study was conducted on patients undergoing cemented hip arthroplasty. There were 542 patients in the PTS group and 534 in the CB femoral component group. There were 333 and 285 hemiarthroplasties in the PTS and CB groups, respectively. The mean age of participants was 82 years (SD 8.1). The majority of patients were classified as American Society of Anesthesiologists (ASA) grade III to IV and were female, comprising 71.2% in the PTS group and 74.5% in the CB group. Most patients, 827, underwent hip arthroplasty due to fractures (76.9%). Cognitive dysfunction was present in 27% (n = 142) to 29% (n = 159) of patients. Cox regression analysis was performed to adjust for confounders such as age, sex, ASA grade, and cognitive dysfunction.

**Results:**

The PTS group had a higher rate of periprosthetic fractures (6.5%) compared with the CB group (1.3%) over the study period from November 2011 to December 2015. The reoperation rate for the PTS groups was 9.7% and 5.2% for the CB group, respectively. The dislocation rates were 4.9% for the PTS and 1.3% for the CB group. The periprosthetic joint infection rate was 3.5% in the PTS and 2.0% in the CB group. In the regression model female sex (HR 2.0, 95% CI 1.2 to 3.1), ASA grade (HR 3.2, 95% CI 1.1 to 8.3), cognitive dysfunction (HR 1.9, 95% CI 1.2 to 3.2), and the type of femoral component (PTS vs CB, HR 0.2, CI 0.1 to 0.3) were correlated with outcome.

**Conclusion:**

CB femoral components were associated with a reduction in adverse events compared with PTS in cemented hip arthroplasty in an older population. These findings support the use of CB femoral components in order to improve patient outcomes and minimize complications in selected cases.

Cite this article: *Bone Jt Open* 2026;7(3):316–325.

## Introduction

Cemented polished tapered stems (PTS) used in hip arthroplasty have been shown to be problematic in older and frail populations due to the high rate of periprosthetic femoral fracture (PFF). PFFs are serious and complex complications of hip arthroplasty, often resulting in substantial morbidity and a diminished quality of life.^1^ These fractures have high mortality rates, particularly in older and more vulnerable patient populations,^2,3^ and are associated with increased healthcare costs.^4-7^ The PTS design is characterized by its polished surface and taper-slip mechanism, which allows for controlled subsidence within the cement mantle. In contrast, the cemented composite beam (CB) aims to achieve stable fixation by creating a strong bond between the implant and the cement mantle, so that both act together as a single structural unit. CB femoral components are generally designed with a more anatomical shape and a roughened surface – features that prevent relative motion between the femoral component and the surrounding cement. This provides immediate stability and helps to distribute mechanical load more evenly, reducing the risk of periprosthetic fracture. Both PTS and CB components have shown good long-term results for aseptic loosening.^8-13^ At Danderyd University Hospital in Sweden, a transition from PTS to CB femoral components in 2014 was driven by increasing evidence linking the PTS to a higher incidence of PFFs.^14-16^ The two- and six-year follow-up results of this transitional change have been previously reported.^17,18^ It led to a decreased rate of PFF from 3.3% to 0.4% at two years.^11,18,19^

The present study reports a ten-year follow-up of PFF rates and other adverse events after the routine change from a cemented PTS to a cemented CB, and evaluates whether the change in femoral component design has a learning curve associated with increased complications. Primary outcome was PFF rates, while secondary outcomes included survival, time to reoperation, and complications.

## Methods

### Study design, setting, and participants

**Fig. 1:**
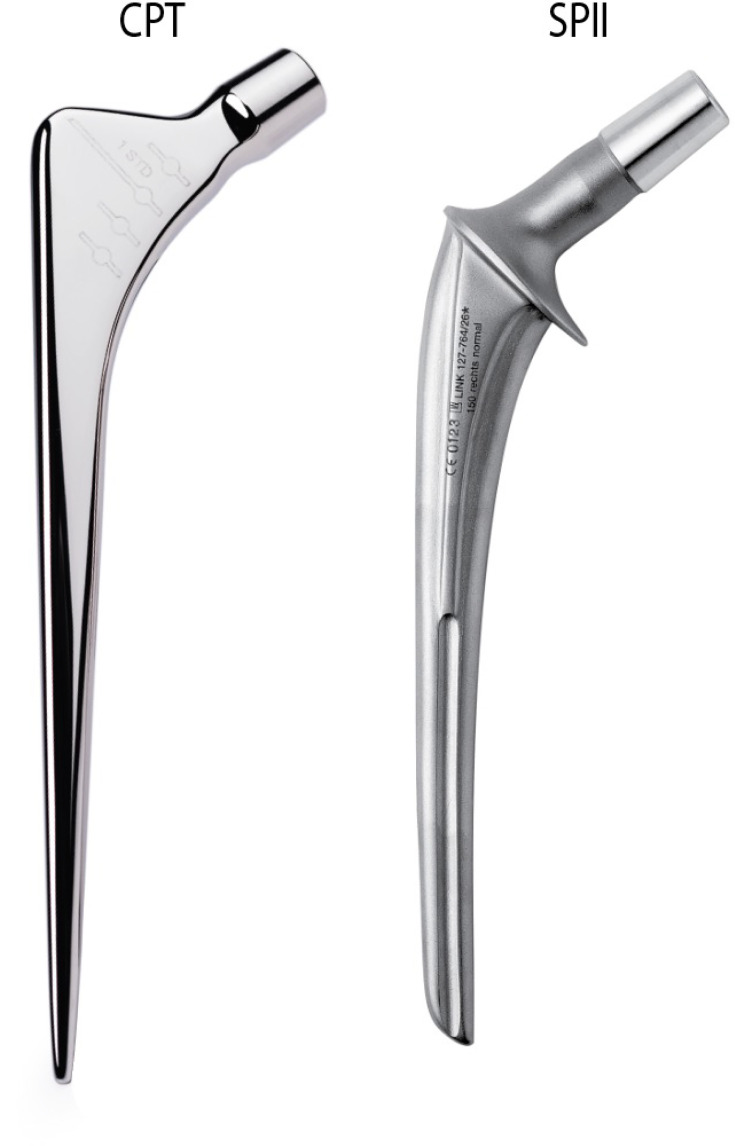
The chrome-based polished tapered stem (CPT; PTS-type femoral component) and the SPII (composite beam (CB)-type femoral component) components used in the study. Images obtained from freely available marketing material from their respective manufacturers.

This study was a long-term, ten-year, prospective observational cohort conducted at the Orthopaedic Department of Danderyd Hospital, Stockholm, Sweden, a university hospital affiliated with Karolinska Institutet serving approximately 600,000 inhabitants. Patients who underwent cemented hip arthroplasty were included, with a Cobalt Chrome-based PTS (CPT; Zimmer, USA) used between November 2011 and December 2013, and the CB (Lubinus SPII; Waldemar Link, Germany) introduced from November 2013 to December 2015 (Figure 1).

The CB fully replaced the PTS in January 2014. The cohort included patients undergoing both hemiarthroplasty (HA) and total hip arthroplasty (THA). Ethical approval was granted by the local review board, with individual informed consent waived for this observational study (DNR: 213/285-31/2).

### Study subjects and descriptive data

**Fig. 2:**
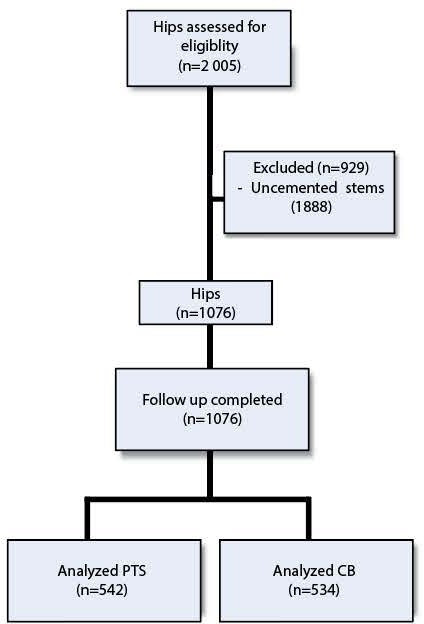
Flowchart of included hips. CB, composite beam; PTS, polished tapered stem.

During the study period, a total of 2,005 hip arthroplasties were performed. After excluding cases involving uncemented femoral components, the final study cohort included 1,076 hips in 1,076 patients (Figure 2).

The baseline demographic characteristics were comparable between the two groups (Table I).

**Table I.** Baseline demographic characteristics.

Characteristic	PTS (n = 542)	CB (n = 534)	p-value*
**Sex, n (%)**			0.152
Male	156 (28.8)	136 (25.5)	
Female	386 (71.2)	398 (74.5)	
Mean age, yrs (SD)	81.5 (8.35)	81.6 (7.99)	0.621
**ASA grade, n (%)**			< 0.001
I to II	136 (25.1)	186 (34.8)	
III to IV	406 (74.9)	348 (65.2)	
Mean BMI, kg/m^2^ (SD)	24.3 (4.16)	24.1 (4.55)	0.423
**Cognitive dysfunction, n (%)**			0.282
No	383 (70.7)	392 (73.4)	
Yes	159 (29.3)	142 (26.6)	
**Type of arthroplasty, n (%)**			0.018
Total hip arthroplasty	209 (38.6)	249 (46.6)	
Hemiarthroplasty	333 (61.4)	285 (53.4)	
**Surgical approach, n (%)**			< 0.001
Direct lateral	411 (75.8)	355 (66.5)	
Posterolateral	131 (24.2)	179 (33.5)	
**Index surgery, n (%)**			0.323
Fracture	432 (79.7)	395 (73.9)	
Degenerative	110 (20.3)	139 (26.1)	

* Chi-squared test.

ASA, American Society of Anesthesiologists; CB, composite beam; HA, hemiarthroplasty; PTS, polished tapered stem; THA, total hip arthroplasty.

### Exposure

Both femoral components were used for hemiarthroplasty (HA) and THA procedures, including both acute cases and elective surgeries. The operations were conducted either by a consultant orthopaedic surgeon or by a registrar under the supervision of a consultant. A direct lateral approach was employed for hip fractures, while a posterolateral approach was predominantly used for elective surgeries. The procedures involved both cemented and uncemented cups, including dual-mobility options. For THAs, head sizes ranged from 22 mm to 36 mm in diameter, whereas a unipolar Cobalt Crome head was used for HAs.

### Variables

We collected data on dates of operation and reoperation, femoral component type, cup type (for THA), patient age, sex, presence of cognitive dysfunction (classified by the treating surgeon; temporary confusion was not considered cognitive dysfunction), American Society of Anesthesiologists (ASA) grade,^20^ indication for surgery, type of arthroplasty (HA or THA), and surgical approach. PFFs were classified according to the Vancouver system^21^ by a senior consultant orthopaedic surgeon (OS). If a patient underwent bilateral operations, either HA or THA, only the first hip was included in the analysis.

### Data sources

We prospectively collected data throughout the study period using each patient’s unique Swedish personal identification number. Data collection involved searches of our hospital’s surgical and medical databases, supplemented by regular follow-up visits. A digital case report form was employed to document relevant details during the study period. Additionally, we consulted the Swedish Hip Arthroplasty Register to detect any reoperations performed at other hospitals; however, no external reoperations were identified. All patients were followed for up to ten years after their primary surgery or until death. Reoperation was defined as any secondary surgical procedure involving the operated hip, including both revisions (exchange or removal of one or more prosthetic components) and other reoperations such as open reduction and internal fixation (ORIF) for periprosthetic fracture. Revision, specifically defined as any procedure involving exchange or removal of a component within the THA/HA construct (femoral component, cup, or liner), was considered a censor event in the analysis. The mean follow-up duration was 5.48 years (SD 2.82) for the CB group and 6.89years (SD 2.42) for the PTS group. This study adhered to the reporting guidelines established by the STROBE (Strengthening the Reporting of Observational Studies in Epidemiology) statement.^22^

### Sample size

The study size for this follow-up study is based on the convenient sample of all patients included in the previous study.^17^ There, a pre-study power analysis determined that a minimum of 431 patients per group would be required to achieve a statistical significance level of 5% and a power of 80%. This calculation was based on the assumption of a PFF rate of 3% in the PTS group compared with 0.5% in the CB group. Given that the orthopaedic department performed approximately 250 to 300 cemented hip arthroplasties annually, the necessary sample size was reached by including all patients who underwent surgery during the two years prior to, and the two years following, the change in implant type.

### Statistical analysis

The covariates included in the multivariable Cox regression model (age, sex, BMI, ASA grade, cognitive dysfunction, surgical indication, and surgical approach) were chosen a priori based on established clinical relevance and previously reported associations with adverse outcomes after hip arthroplasty.^2,3,23^ These factors are well-documented predictors of periprosthetic fracture, revision, or mortality, and were therefore included to reduce the risk of confounding. We analyzed differences in the proportion of hip fractures between the PTS and CB groups using a two-sided Z test. For the primary outcome, we applied a Cox proportional hazards model. Follow-up time was defined as the interval from the date of surgery to the earliest occurrence of death, reoperation (due to PFF, or other revision), or a maximum follow-up duration of ten years. The model was adjusted for the following variables: femoral component type (PTS or CB), age, sex, BMI, ASA grade (I and II vs III and IV), cognitive dysfunction, surgical indication (fracture or degenerative joint disease), and surgical approach. Hazard ratios (HRs) with 95% CIs were used to present the results. The proportional hazards assumption was tested using the Grambsch and Therneau method, analyzing scaled Schoenfeld residuals. A threshold of p<0.05 was considered significant. Demographic data were compared using the chi-squared test. All statistical analyses were conducted using R software, version 4.2.5 (R Foundation for Statistical Computing, Austria).

## Results

### Reoperations

The overall reoperation rate, defined as any secondary surgical procedure involving the operated hip was 12.9% (n = 70) in the PTS group and 5.2% in the CB group (p < 0.001, chi-squared test). The revision rate, defined as exchange or removal of one or more prosthetic components, was 9.7% in the PTS group and 5.2% in the CB group (p < 0.001). PFFs and dislocation were the most common reasons for reoperation. Approximately half of the PFFs in the PTS group were treated with ORIF without component revision, whereas all PFFs in the CB group were managed with ORIF only. PTS patients also experienced higher rates of dislocation (4.1% vs 1.3%) and periprosthetic joint infection (3.0% vs 2.0%) compared with the CB group. The median time from primary surgery to PJI was 28 days (IQR 18 to 46) (Table II). The improved outcomes of the CB group were not influenced by a learning curve, as the complication rates in the first 100 CB cases were comparable to later cases. There were no intraoperative PFFs.

**Table II.** Reasons for reoperation following primary surgery between polished tapered stem (PTS) and composite beam (CB) components.

Reason for reoperation	Overall		Fracture		Degenerative	
	PTS (n = 542)	CB (n = 534)	PTS (n = 432)	CB (n = 395)	PTS (n = 110)	CB (n = 139)
Periprosthetic joint infection	15	11	9	7	6	4
Periprosthetic femoral fracture	33	7	25	4	8	3
Periprosthetic acetabular fracture	0	1	0	0	0	1
Dislocation	21	7	13	5	8	2
Loosening/osteolysis (femoral component)	0	1	0	1	0	0
Other reasons	1	1	1	1	0	0
Any reason for open reoperation (excluding closed reductions)	49	21	35	13	14	8
Any reason for reoperation including closed reductions	70	28	48	18	22	10
**Vancouver classification**						
A	2	2	2	2	0	0
B1	4	0	3	0	1	0
B2	20	0	16	0	4	0
B3	1	0	1	0	0	0
C	6	4	3	2	3	2

**Fig. 3:**
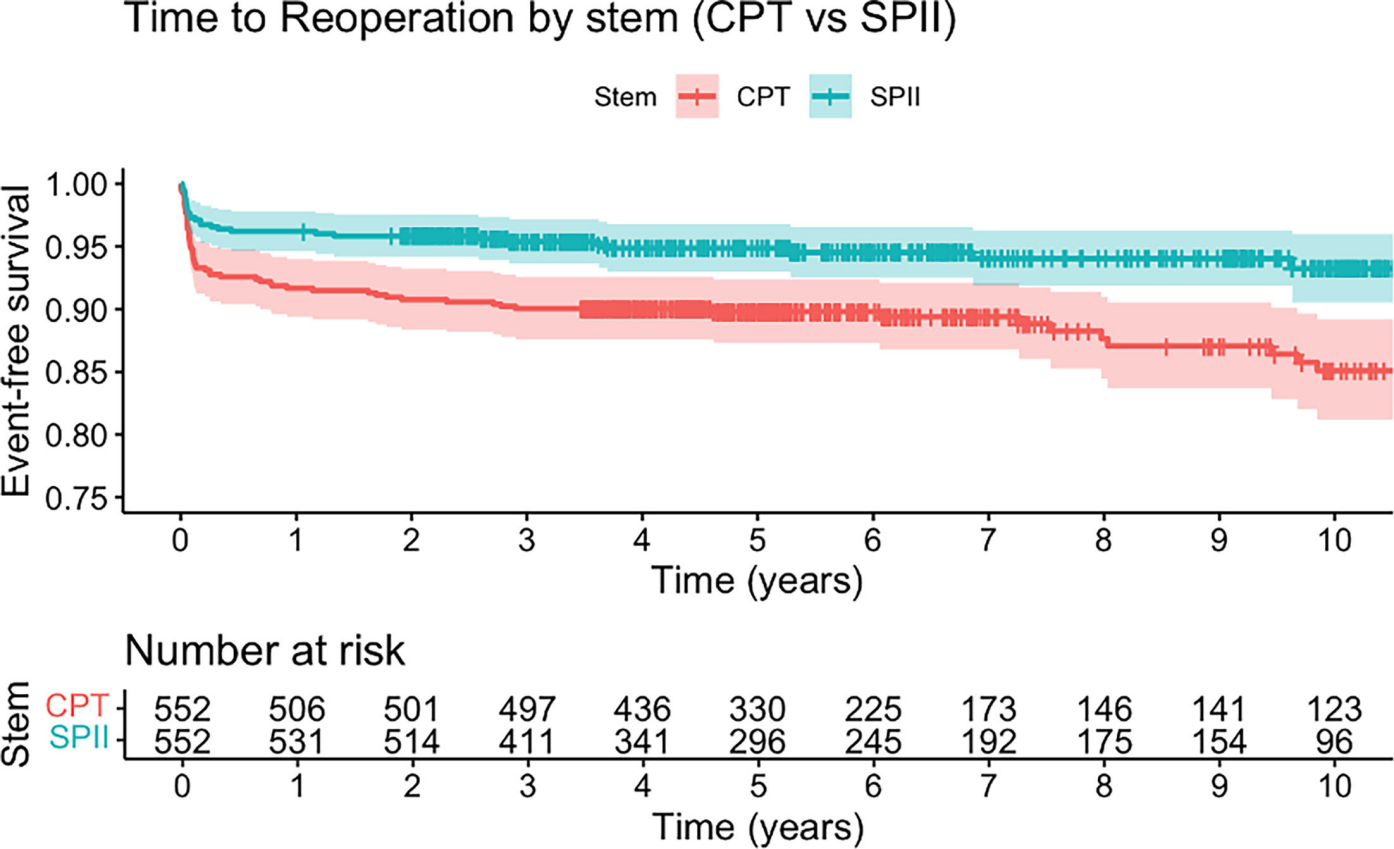
Kaplan-Meier curves for time to reoperation by stem type (chrome-based polished tapered stem (CPT) vs SPII). The figure shows unadjusted Kaplan-Meier survival estimates for time to reoperation within ten years after primary total hip arthroplasty using either a CPT or SPII stem. Shaded areas indicate 95% CIs. The number of patients at risk at each year is displayed below the plot. A log-rank p-value is shown for the comparison between groups.

CB-type femoral components were associated with a lower risk of reoperations compared with PTS-type femoral components (Figure 3) (adjusted HR 0.2, 95% CI 0.1 to 0.3). A significantly shorter time to PFF was observed in the SPII group with 6.08 (SD 3.22) compared with the CPT group 6.8 (SD 3.08) (p < 0.001, chi-squared test).

Females had a significantly higher risk of reoperations compared with males (adjusted HR 2.0, 95% CI 1.2 to 3.1). Higher ASA grade (III to IV) and cognitive dysfunction was also associated with more reoperations (adjusted HR 3.2, 95% CI 1.1 to 8.3) (Table III).

**Table III.** Variables associated with increased risk of reoperations due to periprosthetic femoral fractures (PFFs).

Variable	Total	PFF, n (%)	Crude HR (95% CI)	Adjusted HR (95% CI)
Age			1.0 (1.0 to 1.0)	1.0 (1.0 to 1.0)
BMI			0.9 (0.9 to 1.0)	0.9 (0.8 to 1.0)
**Sex**				
Female	794	24 (3)	1.0 (ref)	1.0 (ref)
Male	282	14 (5)	2.1 (1.1 to 4.1)	2.0 (1.1 to 3.9)
**ASA grade**				
I to II	329	9 (3)	1.0 (ref)	1.0 (ref)
III to V	747	29 (4)	2.2 (1.1 to 4.6)	1.6 (0.7 to 3.4)
**Cognitive dysfunction**				
No	758	29 (4)	1.0 (ref)	1.0 (ref)
Suspected	203	2 (1)	0.5 (0.1 to 2.0)	0.4 (0.1 to 1.7)
Yes	108	7 (6)	2.9 (1.1 to 6.7)	2.6 (1.1 to 6.2)
**Indication for surgery**				
Degenerative	254	10 (4)	1.0 (ref)	1.0 (ref)
Fracture	822	28 (3)	0.7 (0.3 to 1.4)	1.0 (0.5 to 2.2)
**Type of femoral component**				
PTS	542	33 (6)	1.0 (ref)	1.0 (ref)
CB	534	5 (1)	0.2 (0.1 to 0.4)	0.2 (0.1 to 0.4)
**Type of arthroplasty**				
Hemi	640	21 (3)	1.0 (ref)	1.0 (ref)
Total	436	17 (4)	0.9 (0.7 to 1.2)	0.9 (0.8 to 1.1)

ASA, American Society of Anesthesiologists; CB, composite beam; HR, hazard ratio; PTS, polished tapered stem.

**Fig. 4:**
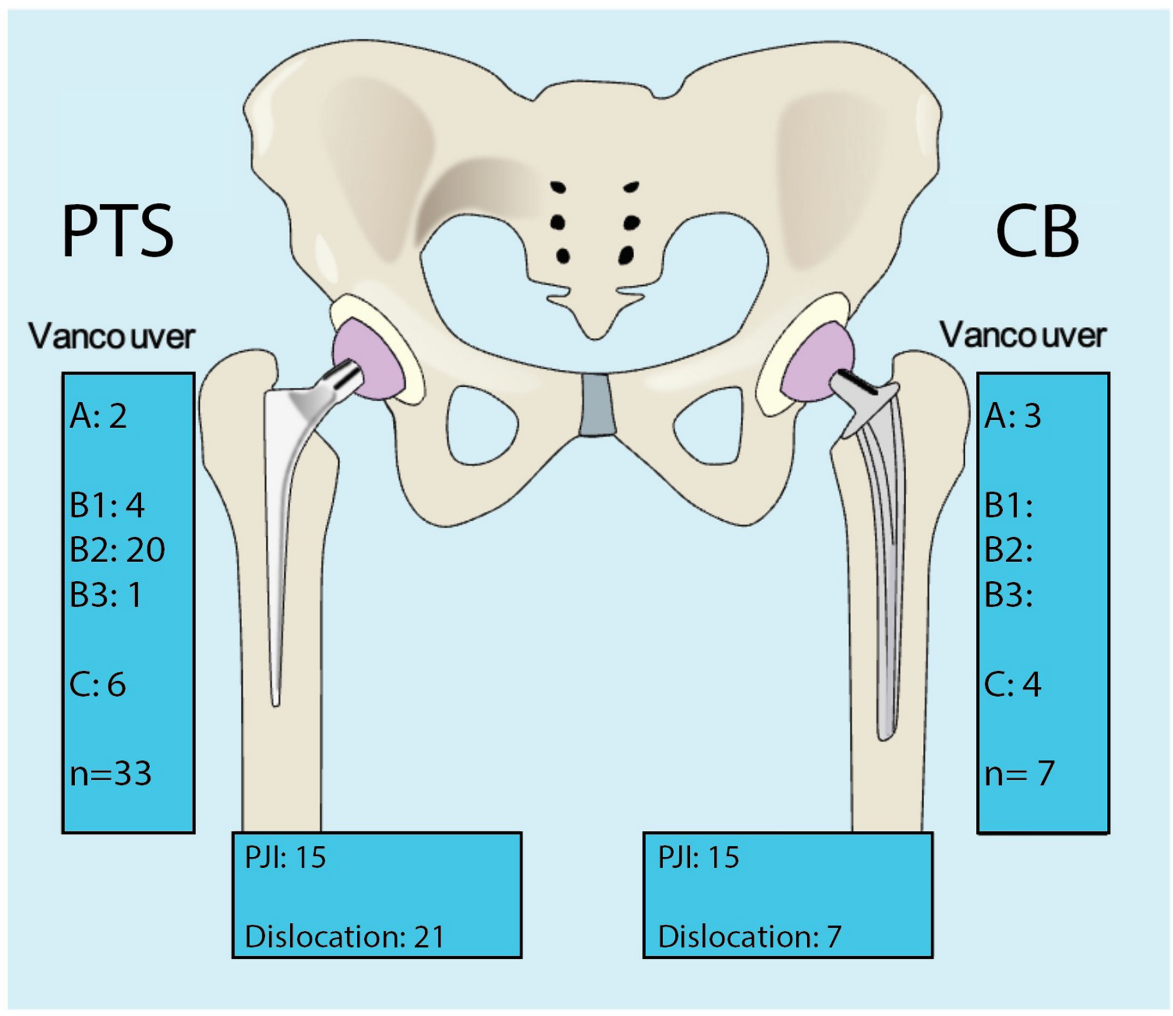
Summary of adverse events in the polished tapered stem (PTS) and composite beam (CB) groups. PJI, periprosthetic joint infection.

The Vancouver grade of PFFs differed between the two groups (Figure 4). PFFs in the PT group included all Vancouver classes, with B2 being the most common. Most of the PFFs in the PTS groups were treated using ORIF (n = 23) while a minority underwent femoral component revision (n = 10). All CB PFFs were treated using ORIF.

The complication rate following treatment for PFF was higher in the PTS group compared with the CB group (Table IV).

**Table IV.** Complications following surgical treatment of periprosthetic femoral fractures.

Variable	PTS (n = 505)	CB (n = 534)
PFF	33	7
**PFF treatment**		
ORIF	23	7
Revision	10	0
**PJI after PFF**	3	0
ORIF	3	0
Revision	0	0
**Dislocation after PFF**	4	0
ORIF	4	0
Revision	0	0

CB, composite beam; ORIF, open reduction and internal fixation; PFF, periprosthetic femoral fracture; PJI, periprosthetic joint infection; PTS, polished tapered stem.

### Mortality and reoperations

**Fig. 5:**
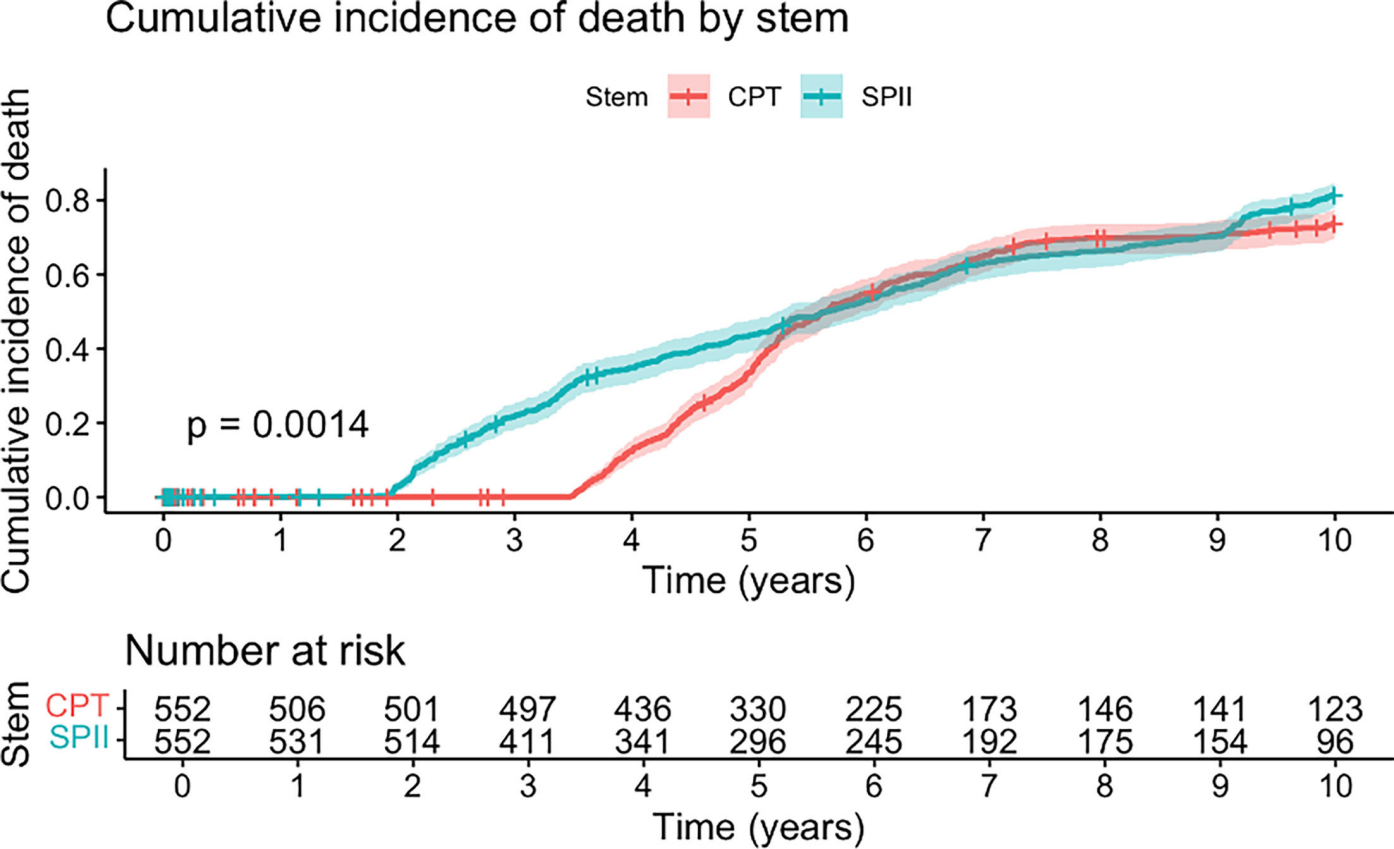
Cumulative incidence of death within ten years after primary total hip arthroplasty by femoral component type. The plot illustrates cumulative incidence (1 – survival) of death within ten years after surgery, stratified by femoral component type (chrome-based polished tapered stem (CPT) vs SPII). Patients who underwent revision were censored at the time of that event. Shaded areas represent 95% CIs, and the number at risk at each year is shown below the figure.

Median follow-up time was 5.5 years for the CB group (IQR 3.6 to 7.5) and 6.9 years (IQR 5.3 to 8.6) for the PTS group (p = 0.002, chi-squared test). The ten-year mortality rate in the cohort was 87.2% (n = 878). No significant difference in mortality was observed between the two groups; the CB group had a median survival of 5.0 (IQR 3.1 to 6.9) and the PTS 4.9 years (IQR 3.3 to 6.6) (Figure 5).

## Discussion

Over a ten-year follow-up period, the transition from PTS to CB was associated with notable reductions in adverse events, particularly PFFs of the Vancouver B classification and dislocations. These findings suggest that CB femoral components are a more advantageous implant system compared with PTS when performing cemented hip arthroplasty in older and frail patient populations.

The main finding of this study was the significant reduction in PFF rates when transitioning to the CB. Similar results have previously been reported in shorter follow-up studies.^18,24,25^ Vancouver A and C fractures occurred at similar rates in both the CB and PTS groups, although Vancouver B fractures were entirely absent in the CB group, whereas they represented the most prevalent fracture pattern in the PTS group. These findings reinforce the notion that the wedge-shaped design of PTS femoral components predisposes patients to metaphyseal split fractures, while the CB design’s uniform engagement with the cement mantle provides better resistance to such fractures.^7,16,26,27^ This distinction is critical because PFFs often lead to poor functional outcomes and require complex surgical interventions, which predispose patients to increased mortality and further complications.^28,29^ PFFs are associated with increased healthcare costs, making their prevention both clinically and economically desirable.^30^

Our findings found a reduction in dislocations and PJIs in the CB group compared with the PTS group. There was no evidence of a learning curve effect or surgeon-specific influence on complication rates. This finding raises important questions regarding the underlying mechanisms responsible for the reduced complication rates observed in the CB group, suggesting that the design and biomechanical properties of the CB femoral component itself might be the primary factors contributing to improved outcomes. It has been suggested that the CB design’s ability to minimize varus alignment and overcorrection of offset may further contribute to its lower dislocation rates.^19,31^

Studies suggest that the CPT femoral component’s design, characterized by its polished and tapered features, contributes to a higher incidence of early PFFs. Brodén et al^6^ found a notably high early PFF incidence when using the CPT femoral component, attributing the risk to the polished tapered design that affects the bone-implant interface. Mukka et al^14^ recommended against using the CPT femoral component for femoral neck fractures in elderly patients, citing a higher prevalence of postoperative PFFs in octogenarians. Erhardt et al^5^ observed femoral component fractures at the CPT level, indicating a vulnerability related to the femoral component’s design. Carli et al^7^ discussed the CPT femoral component’s impact on the cement mantle, leading to Vancouver B2 fractures. Similarly, Khan and McLauchlan^32^ reported higher PFF rates with CPT femoral components in a large cohort of 2,892 patients.

Moreover, Palan et al^33^ found that using CPT femoral components was associated with the highest risk of revisions for periprosthetic fractures when compared with other cemented femoral components. In a similar vein, Scott et al^34^ highlighted the increased early PFF rates with CPT femoral components and stressed caution in their use. A meta-analysis from Mabrouk et al^35^ found that the risk of PFFs is lower in CB compared with PTS femoral components. Moreover, Lamb et al^36^ have also suggested that surgeons should carefully consider femoral component design when choosing implants, with cemented CBs showing the lowest risk for PFFs. However, the cobalt-chrome CPT femoral components seem to be especially prone to PFFs, with a HR at 2.83 compared with Exeter.^36^ Recent research highlights a significant association between the use of the CPT femoral component and an increased risk of PFFs, particularly in elderly patients.^6,23^ The higher susceptibility to fractures in the PTS group may lead to more frequent revision surgeries, which in turn increases the risk of subsequent dislocations and infections.^37^ A particularly complex case could involve a PFF initially managed with a primary revision for fracture fixation, followed by instability and dislocation requiring a secondary revision, which is then complicated by infection, necessitating further revisions.

It is important to note that the PTS group had higher ASA grades and a greater degree of cognitive impairment, which may have influenced the outcomes. However, cognitive impairment did not reach statistical significance. Higher ASA grades are associated with increased risk of reoperation due to PFF (Table III). No changes were made to surgical protocols during the study period, such as cement type, ventilation systems, or antibiotic prophylaxis regimens. Regardless of the underlying mechanisms, the results indicate that transitioning to CB femoral components may mitigate the three most dreaded complications of hip arthroplasty.

The results of our study provide a reasonable argument for the adoption of CB femoral components in cemented hip arthroplasty for older patients at high risk of complications. However, further research is warranted to explore the underlying mechanisms driving these observed benefits, as well as to evaluate the performance of CB in younger, more active populations. Long-term studies assessing implant survivorship, functional outcomes, and patient-reported quality of life would be essential to confirm the durability and broader applicability of our findings.

The strengths of this study lie in its sample size, comprehensive follow-up, and use of national and institutional registries to ensure data accuracy. However, some limitations must be acknowledged. The observational design introduces potential confounders, such as surgeon experience, patient comorbidities, and variations in perioperative care. Although statistical adjustments were made for several key variables, residual confounding cannot be fully excluded. Therefore, causal inferences should be made with caution. Additionally, the generalizability of these findings is primarily limited to older and frail patients.

In conclusion, the transition from PTS to CB femoral components in cemented hip arthroplasty was associated with a significant reduction in adverse events, in particular Vancouver B fractures. Our findings underscore the critical role of implant design in determining the safety and efficacy of hip arthroplasty. This study provides evidence supporting the use of CB over PTS in the selection of femoral femoral components for cemented hip arthroplasty in older and frail patients.

**Take home message**

- Composite beam femoral components reduce the risk of reoperation compared with polished tapered femoral components in cemented hip arthroplasty.

## Data Availability

The dataset used and analyzed during the current study are available from the corresponding author on request.
